# QuickStats

**Published:** 2014-10-24

**Authors:** FROM THE NATIONAL CENTER FOR HEALTH STATISTICS

**Figure:**
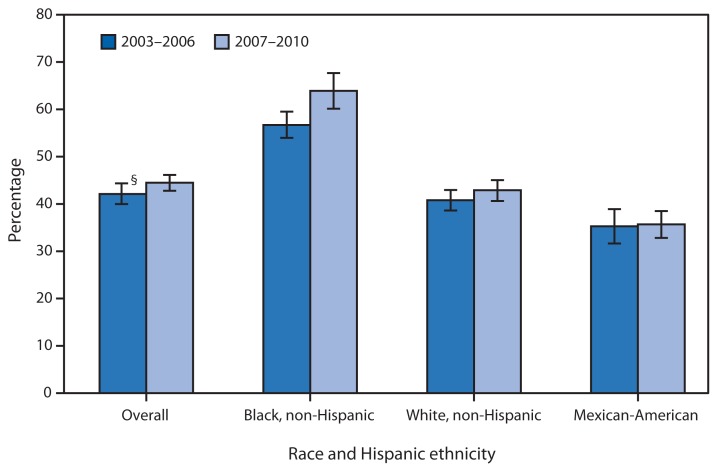
Percentage of Adults Aged 18–59 Years Who Were Ever Tested for Human Immunodeficiency Virus (HIV),^*^ by Race and Hispanic Ethnicity — United States, National Health and Nutrition Examination Survey, 2003–2006 to 2007–2010^†^ ^*^ Based on response to the question, “Except for tests you may have had as part of blood donations, have you ever had blood tested for the AIDS virus infection?” ^†^Statistical significance determined by t-test (p<0.05) ^§^95% confidence interval.

Approximately 44% of adults aged 18–59 years had ever been tested for HIV (other than blood donations) during 2007–2010, nearly the same as during 2003–2006. From 2003–2006 to 2007–2010, no significant change was observed for non-Hispanic white and Mexican-American adults in this age group. A significant increase was observed in the percentage of non-Hispanic black adults aged 18–59 years (from 57% to 64%) who had ever been tested for HIV. During both periods, non-Hispanic black adults had a significantly higher prevalence of any lifetime HIV testing compared with non-Hispanic white and Mexican-American adults.

**Source:** Woodring JV, Kruszon-Moran D, Oster AM, McQuillan GM. Did CDC’s 2006 revised HIV testing recommendations make a difference? Evaluation of HIV testing in the U.S. household population, 2003–2010. J Acquir Immune Defic Syndr 2014;67:331–40.

**Reported by:** Joseph V. Woodring, DO, jwoodring@cdc.gov, 301-458-4599; Deanna Kruszon-Moran, MS; Geraldine M. McQuillan, PhD; Alexandra M. Oster, MD; Steven M. Frenk, PhD.

